# Health related quality of life in patients with anogenital warts

**DOI:** 10.1186/1477-7525-9-67

**Published:** 2011-08-16

**Authors:** Sotirios A Koupidis, Electra Nicolaidou, Maria Hadjivassiliou, Stefanos Bellos, Petros Skapinakis, Christina Stefanaki, Helen Papadogeorgakis, Andreas Katsambas

**Affiliations:** 1 Sexually Transmitted Infections Unit, 1st Department of Dermatology and Venereology, University of Athens, "Andreas Sygros" Hospital, Athens, Greece; 2 Department of Psychiatry, Medical School, University of Ioannina, Greece

## Abstract

**Introduction:**

The health-related quality-of-life (HRQoL) instruments are an important tool for the evaluation of medical outcomes. Sexually transmitted diseases (STDs) influence the patients' life. We aimed to evaluate the HRQoL in patients with anogenital warts at the time of and 1 month after the diagnosis.

**Materials and methods:**

We used the short-form (SF)-36 questionnaire to compare the HRQoL of 91 patients with anogenital warts to 53 control subjects with the same socioeconomic characteristics.

**Results:**

There was no statistical difference in the overall HRQoL measurement between the anogenital wart patients and controls. However, there was an improvement in the scales of vitality (65.22 ± 15.70 vs. 69.04 ± 14.11, respectively; p < 0.05) and mental health (65.00 ± 20.09 vs. 69.43 ± 18.08, respectively; p < 0.05) in anogenital warts patients between the time of diagnosis and 1 month later. Furthermore, there was a significant deterioration in the scale of social functioning (73.47 ± 22.18 vs. 72.89 ± 19.28, respectively; p < 0.05). The small sample size is a limitation of our study.

**Conclusions:**

HRQoL does not appear to be influenced in anogenital wart patients, as measured by the generic instrument SF-36. It is therefore important to develop specific instruments for the measurement of HRQoL in this group of patients.

## Introduction

Sexually transmitted diseases (STDs) are a group of diseases that are transmitted through sexual intercourse and are caused by a wide variety of pathogenic micro-organisms. Until this day, more than 50 micro-organisms have been recognised as a cause of STDs [1]. These diseases comprise a global challenge for health care systems [2-4]. As STDs have reached epidemic dimensions, they are recognized as a considerable threat for public health. In addition, STDs are a cause of acute illness, infertility, disability and death, with serious medical and psychological consequences for millions of people. The STD epidemic is associated with several political, socioeconomic, behavioural, biochemical and biomedical factors.

According to the World Health Organization and Eurostat, there are 28 million new STD cases in Europe per annum [3,4]. The highest incidence is observed in urban populations between 15-35 years of age [3,4].

Skin diseases may have a considerable effect on the patient's quality-of-life (QoL). In 20-50% of patients with skin diseases in secondary care, the decrease in the QoL may be severe enough to classify the patient at risk of developing severe psychosocial impairment or psychiatric morbidity such as clinical depression [5-7].

Human papillomavirus (HPV) infection is the most common cause of STDs worldwide with 50% of the cases involving individuals aged 15-25 years [8,9]. More than 100 different types of HPV have been identified [10], 30 or 40 of which can infect the mucosa and skin of the anogenital area [11,12]. Clinically, anogenital warts consist of epidermal and dermal papules or nodules on the perineum, genitalia, crural folds and anus. They vary in size and can form large, exophytic (cauliflower-like) masses, especially in the moist environment of the perineum. Discrete 1-to 3- mm sessile warts may occur on the penile shaft. Warts may extend internally into the vagina, urethra and perirectal epithelium [1].

The health-related QoL (HRQoL) is very important for the evaluation of medical outcomes. It is measured with generic and disease-specific instruments. These instruments are more important in fields such as Dermatology where mortality is a relatively rare outcome. There is only 1 specific instrument of measuring HRQoL in patients with anogenital warts [13], which has neither been properly adjusted, nor has it been widely translated to be clinically applied.

In the present study we aimed to evaluate the HRQoL in patients with anogenital warts at the time of diagnosis and at one-month follow-up using the generic instrument short-form (SF)-36 [14,15].

## Materials and methods

Between March and October 2008, a total of 240 patients diagnosed for the first time with anogenital warts in the Sexually Transmitted Infections Unit of the "Andreas Sygros" Hospital for Skin and Venereal Diseases were asked to participate in the study. The study consisted of completing the SF-36 questionnaire both at the time of the diagnosis (t_0_) and 1 month later (t_1_). A total of 53 healthy individuals who visited the hospital for acquiring a health certificate comprised the control group. The study participants comprising the control group filled in the questionnaire only once.

The SF-36 is a generic, self-administered, multi-item questionnaire measuring HRQoL, which is widely used in health services research. It consists of 8 scales: Physical Functioning (PF), Role limitations due to Physical problems (RP), Bodily Pain (BP), General Health (GH), Vitality (V), Social Functioning (SF), Role limitations due to Emotional problems (RE) and Mental Health (MH) [16-18]. Each scale ranges between 0 (worst health) and 100 (best health). Furthermore, we compared these results with the scores of SF-36 in patients with other diseases as well as in the general Greek population, where this questionnaire has already been adapted and evaluated [19-21].

This Ethical Committee of the Athens Hospital for Skin Diseases "Andreas Sygros" approved of the study. A written informed consent was obtained from all patients at the time of study entry.

The SF-36 scales were scored according to the documented procedures [16]. Higher scores indicate a better HRQoL. All the statistical analyses were performed with STATA S/E 9.2. A p-value < 0.05 was considered as showing significant results.

## Results

**Figure 1:**
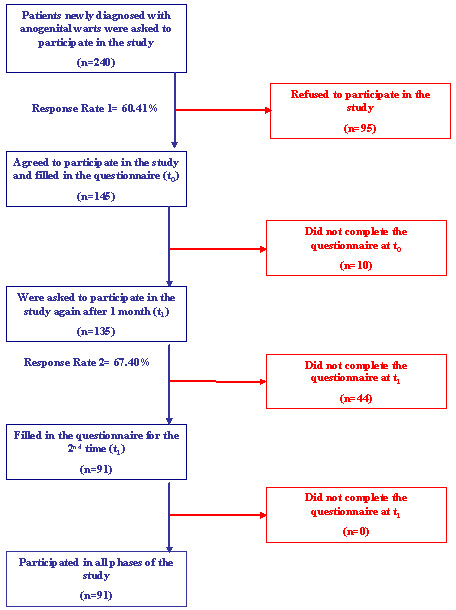
**Flow chart of patients during the study**.

A total of 91 patients completed the study. The dropouts and response rates are presented in Figure 1.

The sociodemographic characteristics of the study participants are presented in Table 1. The results of the 8 scales of SF-36 between the two time intervals in patients with anogenital warts and in the control group are presented in Table 2. In this Table, the scores of the 8 scales for the general population in Greece were also included.

**Table 1:** Socio-demographic characteristics of the study participants

Socio-demographic characteristics of the study participants		
	**Patients (n = 91)**	**Controls (n = 53)**

**Age, Mean**	26.4	26.3

**[95% Confidence Interval]**	[25.0 - 27.9]	[24.5 - 28.2]

**Gender %**		

**Male**	73	74

**Female**	27	26

**Age group %**		

**< 20**	7.7	9.4

**20-29**	71.4	67.9

**30-39**	14.3	17.0

**40-55**	6.6	5.7

**Marital status %**		

**Married**	89.01	88.68

**Single**	6.59	5.66

**Divorced**	3.30	5.66

**Widowed**	1.10	0

**Education %**		

**Primary**	1.10	1.89

**Secondary**	47.25	33.97

**University**	38.46	49.05

**MSc/PhD**	8.79	13.20

**No answer**	4.40	1.89

**Household income %**		

**< 500€**	14.29	9.43

**501€ - 1000€**	30.77	26.41

**1001€ - 2000€**	23.08	33.96

**2001€ - 3000€**	9.89	9.43

**3001€ - 5000€**	8.79	16.99

**< 5001€**	8.79	1.89

**No answer**	4.40	1.89

**Working status %**		

**Working**	61.54	62.26

**Not working (unemployed, student, housewifery)**	36.26	35.85

**No answer**	2.20	1.89

**Table 2:** HRQoL measured with - SF-36

HRQoL measured with - SF-36					
**Scales**	**Patients** **(n = 91, t_0_)**	**Patients** **(n = 91, t_1_)**	**Controls** **(n = 54)**	**General population of Greece (n = 1007)**	

	**Score**				

**Physical Functioning (PF)**	88.72 (SD: 16.63)	91.57 (SD: 14.89)	88.39 (SD: 13.32)	79.5 (SD: 26.3)	NS

**Bodily Pain (BP)**	82.56 (SD: 20.35)	83.34 (SD: 22.27)	78.11 (SD: 23.35)	72.4 (SD: 31.9)	NS

**General Health (GH)**	64.22 (SD: 15.83)	66.36 (SD: 15.06)	70.66 (SD: 18.02)	66.7 (SD: 23.8)	NS

**Role Physical (RP)**	82.78 (SD: 28.21)	83.14 (SD: 26.78)	84.43 (SD: 25.11)	78.6 (SD: 38.7)	NS

**Role Emotional (RE)**	81.11 (SD: 32.79)	83.13 (SD: 28.92)	83.64 (SD: 28.95)	81.2 (SD: 36.6)	NS

**Vitality (V)**	**65.22** **(SD: 15.70)**	**69.04** **(SD: 14.11)**	**68.68** **(SD:15.25)**	**66,0** **(SD: 22.5)**	**p < 0.05**

**Social Functioning (SF)**	**73.47** **(SD: 22.18)**	**72.89** **(SD: 19.28)**	**75.70** **(SD:21.98)**	**81,3** **(SD: 28.7)**	**p < 0.05**

**Mental Health (MH)**	**65.00** **(SD: 20.09)**	**69.43** **(SD: 18.08)**	**66.22** **(SD:19.43)**	**68,2** **(SD: 21.2)**	**p < 0.05**

NS: not significant.

There was no difference in the scores of SF-36 between the patient and the control groups. In 5 of the 8 scales (PF, BP, GH, RP and RE) there was also no significant difference in the 2 consecutive measurements of the patient group. In contrast, there was a slight but significant improvement in V and MH in the patient group between the 2 time intervals. Furthermore, there was a slight but significant deterioration in SF in the patient group between the 2 time intervals.

## Discussion

Our study showed that patients with anogenital warts show an improvement in vitality and mental health 1 month after the establishment of the diagnosis. The possible reasons for this improvement may be the clinical improvement or the clearing of the lesions after 1 month of treatment and the familiarity with the disease. In contrast, our patients showed a slight but significant deterioration in social functioning. This may be the result of feelings of guilt or shame for their condition resulting to avoidance/restriction of social contacts. According to the literature, patients with anogenital warts suffer anxiety about the effect of the disease on their sexual [13,22-27] and social relationships [13,23], the stigma of having contracted a venereal disease [25,27], the uncertain treatment success and time to cure [13,22,23,26] and transmission of the disease to others [13,22-25]. Several studies report that the negative psychological effects of the disease are the most difficult to treat [23,25,27]. They include feelings of anger, fear caused by the relationship of HPV to cervical cancer, guilt, depression, self-loathing and worries about the future [13,22-29]. Finally, the literature points to an increased need for more information about the disease and an improved communication between physicians and patients [22,23,25,27].

Μore male than female patients were recruited in our study. This is because more male patients with STDs seek help for their condition in a hospital for Skin and Venereal Diseases, like ours. Most women consult their gynecologist for conditions like STDs. Furthermore, the small number of women who attended our hospital did not wish to participate in the study.

Our study has some limitations. Firstly, the sample size was relatively small. Nevertheless, it was adequate to reach significant conclusions. In addition, the population of our study was patients attending a public, specialized hospital; it did not include patients from general hospitals, as well as patients from private practitioners. It also did not include more wealthy patients who usually prefer private hospitals to maintain their anonymity, as well as those women who are treated by other specialists (e.g. gynecologists). Therefore one should not generalize the results in such groups if patients.

Based on the above-mentioned arguments, a credible questionnaire/tool is required to measure psychological burden on patients with anogenital warts. The development of a specific questionnaire for measuring HRQoL in patients with anogenital warts, as described by Badia and associates [13], could demonstrate the degree of psychological/social/physical burden of this condition to patients. In addition, use of this questionnaire in different populations (i.e. with different religions, ethical and social beliefs, etc.) will help in drawing conclusions about the relative burden of the condition according to the patients' background.

There are several clinical implications of our study. Firstly, by use of the questionnaire, patients are directly involved and may participate in their treatment more actively. This way the emphasis is shifted from disease-oriented to patient-oriented treatment. For this reason assessment of the HRQoL has been routinely used as a measure of efficacy in clinical practice and research [30]. Additionally, there is the potential for physicians to receive feedback from the patients regarding the effectiveness of the treatment and their degree of satisfaction. Furthermore, the use of the SF-36 in specific patient subgroups with different religious or cultural background compared to the rest of the Greek population (e.g. Muslims, refugees, etc.) may have a different effect on HRQoL. Finally, the employment of these tools will enable the more objective verification of the success or failure of the therapeutic approach and will also implicate the patient himself/herself in the management of the condition.

## Conclusions

In conclusion, we found that there was an improvement in the scales of vitality and mental health and a deterioration in the scale of social functioning in anogenital warts patients between the time of diagnosis and initiation of treatment and one month later. The small sample size however is a limitation of our study. HRQoL does not appear to be influenced in anogenital wart patients, as measured by the generic instrument SF-36. It is important to develop specific instruments for the measurement of HRQoL in this group of patients.

## Conflict of interest

The authors declare that they have no competing interests.

## Authors' contributions sections

SK participated in the design of this ancillary work, reviewed the literature. He also participated in generating and gathering the data the data and in writing the manuscript. EN participated in generating the data and in writing the manuscript. MH participated in the design of the study and in writing the manuscript. SB Performed the statistical analysis, and drafted the manuscript. PS made critical comments and helped in the interpretation of the results. CS participated in generating and in gathering the data of the study. HP participated in generating and gathering the data of the study. AK participated in the design and coordination of the study. All authors collaborated interactively, and read and approved the final version.
